# Radiotherapy in Combination with CD20×CD3 Bispecific Antibodies for B-Cell Lymphoma: Biological Rationale, Current Evidence, and Open Questions

**DOI:** 10.3390/cancers18172895

**Published:** 2026-09-07

**Authors:** Patrizia Ciammella, Alberto Bavieri, Maria Elena Nizzoli, Emanuele Alì

**Affiliations:** 1 Radiation Oncology Unit, Azienda Unità Sanitaria Locale-IRCCS di Reggio Emilia, 42122 Reggio Emilia, Italy; emanuele.ali@ausl.re.it; 2 Hematology Specialization School, University of Modena and Reggio Emilia, 41121 Modena, Italy; alberto.bavieri@ausl.re.it; 3 Hematology Unit, Azienda Unità Sanitaria Locale-IRCCS di Reggio Emilia, 42122 Reggio Emilia, Italy; mariaelena.nizzoli@ausl.re.it; 4 Clinical and Experimental Medicine Doctorate School, University of Modena and Reggio Emilia, 41121 Modena, Italy

**Keywords:** bispecific antibodies, radiotherapy, lymphoma, DLBCL, glofitamab, epcoritamab, perspective, tumour flare reaction

## Abstract

**Background/Objectives:** CD20×CD3 bispecific antibodies (BsAbs) have changed the management of relapsed/refractory (R/R) B-cell non-Hodgkin lymphoma. Whether and how radiotherapy (RT) can be safely and usefully combined with BsAb therapy remains undefined. This Perspective reviews the biological rationale and the available clinical evidence, distinguishing the established findings from the hypotheses. **Methods:** We conducted a targeted, non-systematic review of RT combined with CD20×CD3 BsAbs in B-cell lymphoma, searching PubMed/MEDLINE, the Cochrane Library, and ClinicalTrials.gov, supplemented by conference abstracts and a post hoc verification search. Given the immaturity and heterogeneity of the field, we used a narrative synthesis rather than a systematic search. Sources were classified as direct evidence, mixed/indirect (non-disaggregable) evidence, or registered trials without results; only the first category was used to support clinical claims. **Results:** Direct evidence comprises three case reports, a 7-patient lymphoma subgroup, and a 29-patient cohort with disaggregated CRS/neurotoxicity data. Additional mixed/indirect evidence comes from a 12-patient series. Three registered trials have not yet reported results. Five partially overlapping rationales are proposed: cytoreduction, the modulation of the tumour microenvironment, the management of tumour flare, the consolidation of residual disease, and urgent local control during BsAb step-up dosing. The potential antagonistic effects, including CD20 loss and T-cell depletion, have received comparatively little study. Whether RT-induced lymphopenia could blunt BsAb efficacy remains a key open question. **Conclusions:** The available evidence indicates only that RT has occasionally been delivered around BsAb therapy in small, non-comparative series, without a consistent toxicity signal. It does not establish the benefit, synergy, or optimal sequencing. Prospective studies are needed to define the role of RT in this setting.

## Simple Summary

Bispecific antibodies are a new treatment for lymphoma. They redirect a patient’s own immune cells to attack the tumour. Radiotherapy is an established local treatment for cancer. In current practice, the two are sometimes combined, although this combination has not been formally studied. In this Perspective, we review the evidence available on combining radiotherapy with one class of bispecific antibodies (CD20×CD3) in B-cell lymphoma. We describe the biological rationale for the combination, and the mechanisms by which it could instead be counterproductive. Direct evidence is limited to a small number of case reports and small cohorts, and it is discordant: at least one report describes an inflammatory reaction possibly triggered by radiotherapy during treatment. On this basis, the evidence does not yet support practice recommendations. We reframe the proposed clinical scenarios as open research questions, including the theoretical concern that radiotherapy could reduce the immune cells these drugs depend on, and outline what prospective studies would need to demonstrate before this combination could be adopted in routine practice.

## 1. Introduction

CD20×CD3 bispecific antibodies (BsAbs) have changed the management of relapsed/refractory (R/R) B-cell non-Hodgkin lymphoma. Epcoritamab, glofitamab, and odronextamab are approved for R/R DLBCL; mosunetuzumab is approved for R/R follicular lymphoma. These off-the-shelf agents redirect endogenous T cells against CD20-expressing B cells and achieve clinically meaningful response rates without the logistical constraints of cellular therapies. Even in the pivotal trials, however, the response is neither universal nor durable for most patients. Overall response rates of 52–66% (complete response at 36–39%) leave a substantial proportion of patients without disease control. Even among all treated patients, not only non-responders, the 12-month progression-free survival is only 37% with glofitamab, and median progression-free survival is 4.4 months with epcoritamab [1,2,3,4]. This gap, patients who do not respond or whose response is not sustained, is the principal clinical rationale for considering RT. RT is a local treatment that could address residual or progressive disease without compromising the systemic mechanism on which BsAb efficacy depends. Two properties make RT well-suited to this role. Lymphomas are among the most radiosensitive malignancies, with response rates exceeding the 90% reported even at low doses in indolent histologies [5,6,7], although such low-dose regimens are not expected to achieve a comparable, sustained mass reduction in more aggressive histologies such as DLBCL. RT is also delivered locally, avoiding the systemic myelosuppression associated with additional chemotherapy, which could itself deplete the T-cell pool on which BsAb efficacy depends. These properties motivate the clinical scenarios examined in this review. The need is amplified in patients who relapse after CAR-T therapy or require BsAb therapy after multiple prior lines, who often have few remaining options. These clinical and biological considerations motivate the investigation of RT–BsAb combinations.

This review maps the biological rationale, the available direct clinical evidence, and the unresolved, in some cases previously mischaracterised, questions surrounding RT–BsAb integration.

## 2. Materials and Methods

This Perspective addresses the biological rationale for combining radiotherapy with CD20×CD3 bispecific antibodies in B-cell lymphoma, the direct clinical evidence currently available, and how mature that evidence is relative to a practice-guiding standard. Given the scarcity and heterogeneity of the literature, we used a targeted search strategy rather than a systematic- or scoping-review protocol. We did not apply a formal PICOS/PCC framework, protocol registration, PRISMA-type checklist, or formal risk-of-bias synthesis, which would, in any case, imply a level of comparability the source studies do not have. All study designs were considered eligible: case reports, case series, retrospective cohorts, registered trials, and conference abstracts describing RT delivered before, during, or after CD20×CD3 BsAb therapy in patients with indolent or aggressive B-cell lymphoma at any treatment line. BsAb-monotherapy studies were used only as background efficacy/toxicity benchmarks. Reports combining RT with bispecific antibodies directed against non-CD20 targets, such as BCMA or GPRC5D in multiple myeloma, were excluded from the primary synthesis and discussed only as indirect, supportive evidence. Because much of the identified data pools CD20×CD3 outcomes with those of other constructs, chiefly blinatumomab, or other diseases, each source was classified as direct evidence, mixed/indirect evidence, or a registered trial without results; only direct evidence was used to support summary statements on efficacy or safety. Two reviewers (P.C. and E.A.) independently identified and extracted data from all included studies, resolving disagreements by consensus. Searches covered PubMed/MEDLINE and the Cochrane Library (CENTRAL) for studies published in English from January 2017 to July 2026, combining controlled vocabulary and free-text terms for radiotherapy, CD20×CD3 bispecific antibodies (individually and by class), and B-cell lymphoma. Complete database-specific search strings and dates are provided in Supplementary Table S1. Duplicate records were identified by cross-checking titles, DOIs, and PubMed/Cochrane identifiers across the two databases. ClinicalTrials.gov was searched independently, and conference proceedings from ASH, ASTRO, ESMO, and ICML (2023–2026) were screened manually by title. A post hoc verification search in August 2026, prompted by peer review, used the same core terms to identify records published or registered after the formal search date. It identified one additional registered trial (NCT07634289, “Radio-GLO”) and one additional case report describing a CRS-compatible febrile episode after RT during epcoritamab therapy; both are incorporated throughout. This verification does not constitute a systematic multi-database search and cannot exclude further eligible records, including non-English-language reports.

## 3. Results

The database search (PubMed/MEDLINE, Cochrane CENTRAL) yielded 44 unique records. No duplicate records were identified by the cross-checking procedure described above (Section 2). Given the substantial indexing overlap between MEDLINE and CENTRAL, a zero-duplicate yield is atypical; we report this as a limitation of search sensitivity or of the deduplication procedure, rather than as evidence of a comprehensive search (Section 6). Following the title and abstract screening, eight records underwent a full-text assessment. Of these, four met the predefined eligibility criteria: Loap et al. [8], Fujita et al. [9], Ababneh et al. [10], and Strati and Spiotto [11]. The latter is counted as a screened, background-only source, not included in the primary evidence synthesis (Section 2). The remaining four full-text records were excluded for an unrelated bispecific target, non-disaggregable data, overlap with a larger series, or the absence of clinical outcome data. The ClinicalTrials.gov search identified two eligible registered trials at the time of the formal search, NCT07528352 [12] and NCT06867536 [13], both included. The post hoc verification search (Section 2) identified a third trial, NCT07634289 [14], now also included. A manual screening of the conference proceedings identified one additional eligible abstract (Ren et al. [15]). One further eligible study, Baron et al. [16], was identified through manual citation tracking after the formal search date, having been published shortly before submission; we included it given its direct relevance and size, and its blinatumomab-containing courses are reported separately throughout, per the evidence classification framework (Section 2). The post hoc verification search also identified one additional eligible case report, a CRS-compatible febrile episode during epcoritamab therapy after RT [17], now included. In total, six sources of direct or mixed/indirect clinical evidence were identified: three case reports (including the newly identified one above), one real-world series, and two retrospective cohorts with a mixed histology or bispecific agent, together with three registered prospective trials without results and one earlier, broader review, Strati and Spiotto [11], included for background context only. All are summarised in Table 1. All direct evidence is at the case-report or small (≤29-patient), single-arm, retrospective-cohort level, without a comparator arm, a very-low-certainty evidence base by any standard hierarchy, such as GRADE. Every subsequent finding should be interpreted with this limitation in mind.

### 3.1. Biological Rationale

**Figure 1:**
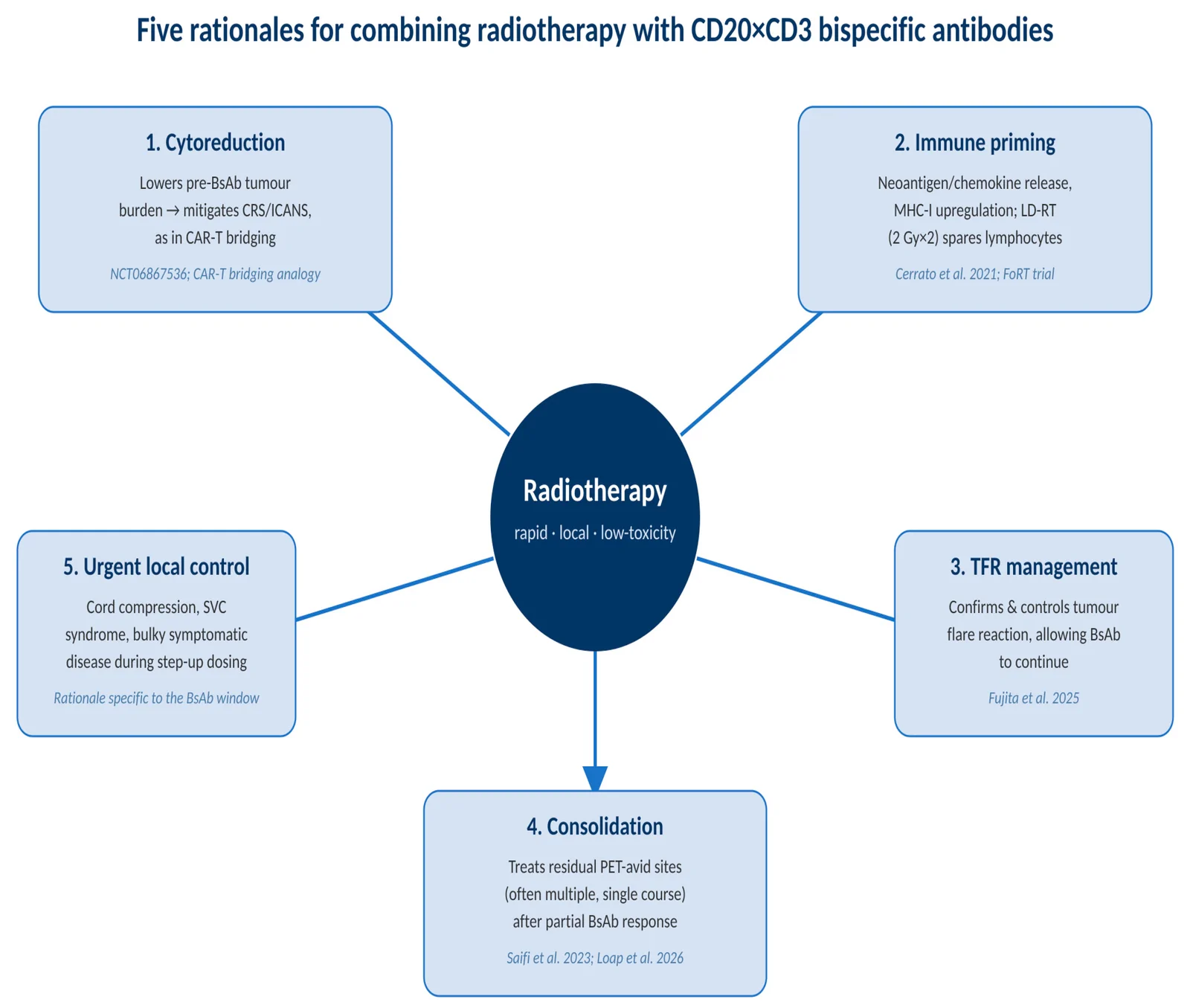
Five hypothesised rationales for combining radiotherapy with CD20×CD3 bispecific antibodies, labelled by evidentiary status [9,17,18,19].

**Figure 2:**
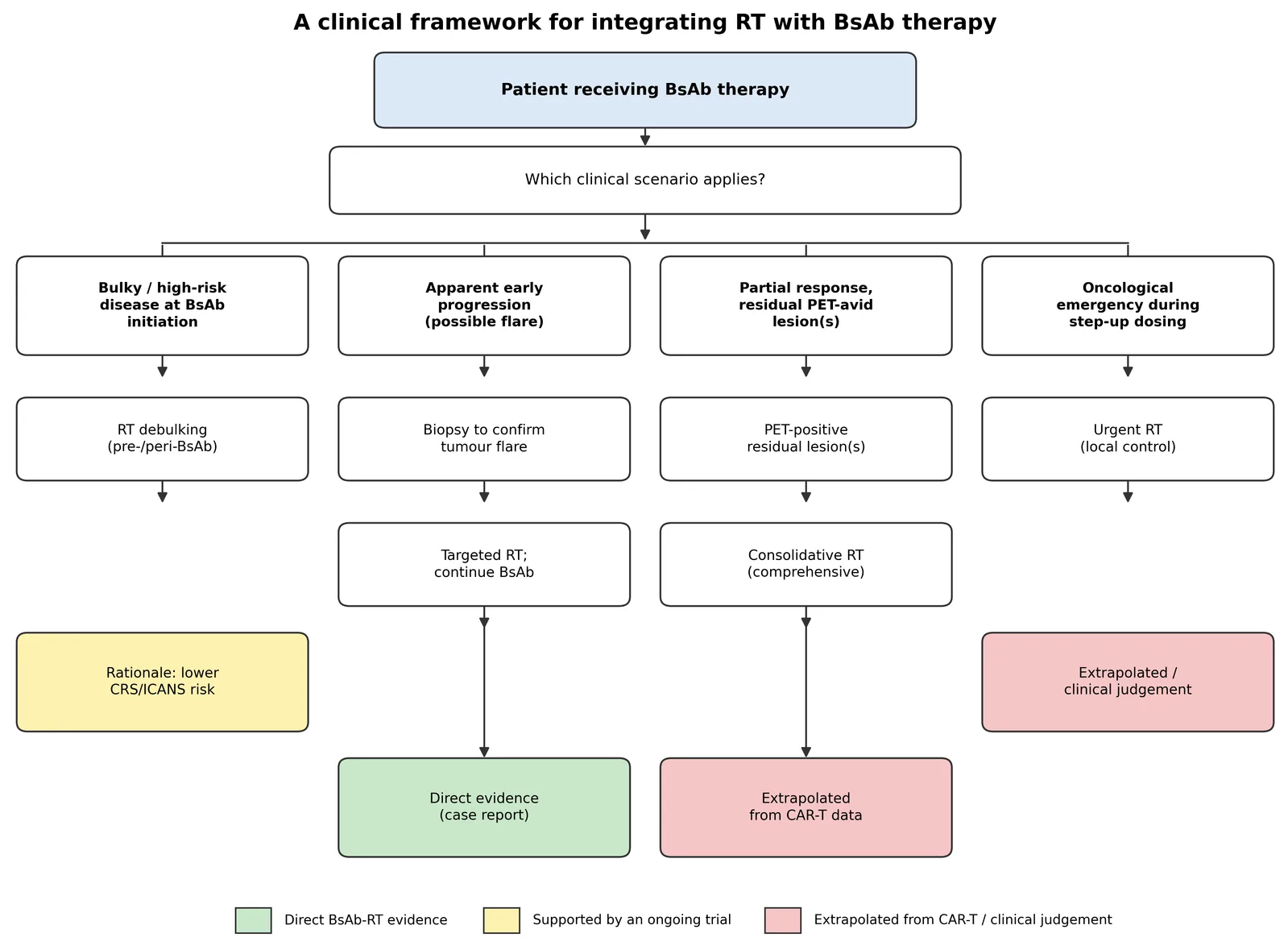
Clinical situations in which RT has occasionally accompanied CD20×CD3 BsAb therapy, reframed as open research questions rather than an actionable algorithm.

The rationale for combining RT with CD20×CD3 BsAbs can be grouped into five partially overlapping, hypothesis-level concepts: cytoreduction, the modulation of the tumour microenvironment, the management of treatment-related phenomena, the consolidation of residual disease, and urgent local control during the BsAb step-up phase. None of these is an established indication. Direct CD20×CD3 clinical evidence exists only for the tumour-flare-reaction scenario, and, even there, the evidence is discordant (see below). The remaining four rationales are preclinical, indirect, or extrapolated from the CAR-T setting. Figure 1 summarises these mechanisms alongside their current evidentiary status; Figure 2 recasts the associated clinical scenarios as open research questions rather than a treatment algorithm.

Cytoreduction (hypothesis; indirect evidence only): In the pivotal trials of glofitamab, epcoritamab, and odronextamab, CRS occurred in roughly half of the treated patients, graded according to the ASTCT consensus criteria [20], although grade ≥ 3 events were uncommon (≤4%) [1,2,3,4]. A validated predictive risk model for glofitamab, and a multicentre real-world analysis of both glofitamab and epcoritamab, identified the baseline tumour bulk as a risk factor for CRS/ICANS [21,22]. This is an association, not a demonstrated causal target of RT. No study identified in this review tested, or was designed to test, whether RT-mediated debulking lowers CRS/ICANS incidence or severity. Reducing the tumour burden before step-up dosing is a biologically plausible hypothesis, but it remains untested. NCT06867536 and the newly identified NCT07634289 are now testing this directly, combining RT (hypofractionated, in the former) with glofitamab in patients with a high tumour burden; neither has reported results.

The modulation of the tumour microenvironment (hypothesis; preclinical/indirect evidence only): RT can induce local inflammatory changes and enhance the antigen presentation, potentially increasing the tumour susceptibility to T-cell engagement by BsAbs. RT-induced immunogenic cell death and T-cell priming, including the abscopal phenomenon, have been characterised in preclinical and clinical settings outside lymphoma [23,24]. Within lymphoma itself, abscopal-like regressions have been documented after involved-site RT in indolent disease [25]. This lymphoma-specific evidence does not involve BsAbs, however, and does not demonstrate synergy with CD20×CD3-mediated immune-synapse formation, T-cell recruitment, or serial killing. We did not identify direct preclinical evidence of RT enhancing CD20×CD3 BsAb activity specifically. Low-dose, lymphocyte-sparing regimens have been proposed as a means of exploiting this effect without the toxicity of higher doses (a working definition of “lymphocyte-sparing RT” is given in Section 3.3), but this remains an unstudied hypothesis in the BsAb setting.

Potential antagonistic biological effects (underexplored): RT could also act against BsAb activity through several plausible mechanisms. These mechanisms have received comparatively little attention to date, not because the evidence favours a net benefit, but because they have been studied less. A reduction in viable tumour cells could reduce the CD20 antigenic target available for T-cell engagement. RT can deplete intratumoral effector T cells and circulating lymphocytes. Damage to the draining lymphoid structures could impair the antigen presentation and T-cell trafficking. RT can induce immunosuppressive myeloid populations and increase TGF-β signalling. Cytoreduction under selective pressure could also favour antigen-low-resistant clones. We are not aware of direct CD20×CD3-specific data addressing any of these possibilities, and we flag this as a priority area for translational study (Section 5).

The management of tumour flare reaction (TFR) (case-level evidence; discordant): This is the only one of the five rationales with direct CD20×CD3 clinical evidence, and that evidence is discordant. Fujita et al. [9] used RT after a biopsy confirmed TFR rather than true progression during epcoritamab therapy, allowing local control of the lesion while epcoritamab continued uninterrupted. The histopathological basis of TFR during epcoritamab therapy has since been characterised in more detail elsewhere [26]. Conversely, a newly identified case report describes a CRS-compatible febrile episode arising after local RT delivered during late-phase epcoritamab therapy, recurring after consecutive fractions and resolving with the IL-6 blockade. This raises the possibility that RT can, itself, act as an inflammatory trigger during BsAb therapy, rather than only as a tool for managing a flare [17]. With two discordant case-level reports, TFR management cannot be established as a validated diagnostic or management scenario. It illustrates an open question, whether and under what circumstances RT during BsAb therapy is more likely to help or to provoke an inflammatory reaction, rather than a proven pathway.

The consolidation of residual disease (hypothesis extrapolated from CAR-T; no direct BsAb outcome data): This rationale borrows from the CAR-T literature: residual FDG-avid disease after T-cell-directed therapy tends to remain radiosensitive, and consolidative RT has produced encouraging results after CAR-T infusion [18,27]. Nothing identified in this review specifically evaluates this indication after BsAb therapy. Baron et al.’s cohort [16] included RT delivered after BsAb therapy in a subset of courses (37%), but this was not specifically characterised as the consolidation of residual PET-positive disease, so direct evidence remains lacking. A further caveat is that the residual PET avidity after T-cell-engaging therapy can reflect inflammatory infiltration or tumour flare rather than viable lymphoma [18]. Histological confirmation, serial imaging, and attention to metabolic-response timing are prerequisites for any consolidative strategy; none of the identified sources addressed this systematically.

Urgent local control (hypothesis/clinical judgement; no direct BsAb-specific data): Step-up dosing is designed primarily to mitigate the CRS risk during BsAb initiation. This does not, by itself, demonstrate an absence of early antitumour activity, and we did not identify direct evidence for a biologically “delayed” therapeutic window. RT remains a plausible tool for urgent, local, immune-status-independent disease control in this setting, but this application currently rests on extrapolation and clinical judgement rather than direct evidence.

### 3.2. Existing Clinical Evidence

For context, in their respective pivotal single-agent trials, glofitamab, epcoritamab, and odronextamab achieved overall response rates of approximately 52–66% (complete response 36–39%) in heavily pretreated R/R DLBCL, with CRS in around half of patients (grade ≥3 ≤4%) [1,2,3,4]. These benchmarks provide the context against which the RT-combination reports below should be interpreted, not a basis for inferring combination effects.

Direct or mixed/indirect clinical evidence on RT combined with CD20×CD3 BsAbs remains limited to three case reports, one small real-world series, one retrospective cohort with a disaggregable CD20×CD3-lymphoma subgroup, one retrospective cohort with CD20×CD3/CD19×CD3 data disaggregable for CRS/ICANS but not for response outcomes, and three registered prospective trials without reported results, summarised in Table 1. We report the outcomes below by endpoint, per the evidence classification framework in Section 2, rather than as an intermixed narrative.

In-field/local response: Loap et al. [8] described the first case of concurrent salvage RT with glofitamab for an early-progressing site; it was well-tolerated, with a complete metabolic response at three months. Among the 44 evaluable lesions in Baron et al.’s cohort [16] (mixed CD20×CD3/blinatumomab courses, not disaggregated for this endpoint), the in-field overall response rate was 84% (complete response 45%). Lymphomas are intrinsically radiosensitive; an in-field response rate of this magnitude following RT cannot, without a comparator, be interpreted as evidence of additive or synergistic activity with a bispecific antibody. It is consistent with, but does not establish, a BsAb-specific contribution.

Out-of-field/systemic response: No source identified in this review reported an out-of-field or systemic response specifically attributable to the RT component; this endpoint was not separately assessed in any identified study.

The duration of local control: This was not systematically reported by any source; the follow-up varied and was frequently short (Table 1).

Progression-free and overall survival: No source reported PFS/OS specifically attributable to, or stratified by, RT exposure.

CRS/ICANS: In Baron et al.’s cohort [16], CRS and ICANS are disaggregated by the BsAb agent. Among CD20×CD3 courses specifically (mosunetuzumab, epcoritamab, glofitamab; *n* = 24), CRS occurred in 6 (25%) and ICANS in 2 (8.3%), consistent with pivotal BsAb monotherapy trials and without an apparent relationship to RT timing or dose. The blinatumomab courses (*n* = 7, a CD19×CD3 construct outside this review’s primary focus) are reported separately (CRS 1/7, ICANS 2/7) and excluded from the CD20×CD3-specific figures above. Set against this, the newly identified case report [17] describes a CRS-compatible febrile episode temporally associated with RT delivered during late-phase epcoritamab therapy. It is a single case, but a concrete counter-example to any claim that RT–BsAb combinations are free of safety signals.

Haematological toxicity/RT-related adverse events: All RT-related toxicities reported by Baron et al. were grade 1–2, with no RT-related hospitalisations [16]. Ababneh et al. reported manageable toxicity regardless of RT sequencing in their LBCL subgroup [10]. These small, retrospective, non-comparative, likely underpowered series cannot exclude uncommon but clinically important toxicities, such as severe CRS/ICANS, prolonged cytopenias, opportunistic infection, or delayed immune suppression. “No observed excess toxicity” in the original literature should be read as “no excess toxicity signal detected in small series,” not as evidence of safety.

Infection: This is not systematically reported by any identified source.

T-cell and lymphocyte changes: Early post-RT lymphopenia was common in Baron et al.’s cohort but did not differ significantly by RT timing relative to BsAb therapy [16]. This is the first empirical data on this question (discussed further in Section 3.4). This single-institution, retrospective observation was not designed or powered to correlate lymphopenia with the BsAb response, however, and none of the identified sources reported functional T-cell subsets (CD3+/CD4+/CD8+), exhaustion markers, or clonality.

Population and exposure details for each source, including the disaggregable LBCL subgroup within Ababneh et al.’s cohort [10] and Baron et al.’s status as the largest dedicated CD20×CD3 series to date [16], are summarised in Table 1a. The heterogeneity of concomitant therapy across sources (RT was one of several concurrent interventions in the Ren et al. series [15]) limits the attribution of outcomes to RT specifically for most sources in this review.

Three prospective trials are now registered to evaluate RT–BsAb combinations directly (Table 1), none with reported results. This body of evidence establishes feasibility, and an absence of uniform harm, in small, selected series. It does not establish the optimal dose, timing, patient selection, synergy, or survival benefit.

### 3.3. Technical Aspects

Because the available evidence remains limited, no standard approach exists regarding the RT sequencing, dose, fractionation, or target volume in combination with BsAbs. No source reported a sufficient set of parameters, such as the field size, dose to circulating blood, nodal/splenic/marrow inclusion, fractionation, baseline lymphocyte count, prior therapy, or corticosteroid exposure, to characterise a reproducible “lymphocyte-sparing” technique; this remains a design principle for future studies rather than an established approach. The RT timing relative to BsAb administration has varied: concurrent (Loap et al. [8]), before BsAb initiation (NCT07528352), and for cytoreduction (NCT06867536, NCT07634289). No source found a significant association between timing and toxicity, response, or lymphopenia [10,16]. The target volume selection is similarly unresolved. Available case reports focused on limited symptomatic lesions, whereas the CAR-T experience suggests the comprehensive irradiation of FDG-avid disease may improve control. This carries plausible costs, such as marrow exposure, lymphopenia, and organ toxicity, against an unproven benefit, particularly since residual PET avidity may reflect inflammation rather than viable lymphoma (Section 3.1). The dose and fractionation remain heterogeneous and under-reported. Toxicity attribution is complicated by the clinical overlap between RT-related inflammation and early BsAb-related events including CRS, as illustrated by the newly identified CRS-compatible fever case [17]. Because BsAb activity depends on endogenous T-cell recruitment, RT-induced lymphopenia deserves particular attention (Section 3.4). Prospective studies with immune monitoring and dosimetric analysis are needed to define whether RT can be integrated without compromising the BsAb efficacy.

### 3.4. Relationship with the CAR-T Paradigm

The rationale for combining RT with T-cell-based therapies in B-cell lymphoma draws, in part, on the experience with CAR-T, which encompasses two distinct scenarios. The first is bridging RT, given before infusion. It is supported by multicentre experience showing excellent local control and favourable outcomes, without synergistic toxicity, alongside subsequent CAR-T [19,28,29,30,31]. Here, however, the parallel with BsAbs is conceptually weak. CAR-T bridging involves intentional lymphodepletion, typically with fludarabine/cyclophosphamide, given specifically to promote the engraftment of the infused cell product, a planned, dosed, fixed-interval intervention with no equivalent in BsAb therapy.

The second scenario is consolidative RT for residual disease after CAR-T infusion. This is the closer parallel to the hypothesis raised in Section 3.1, both involve irradiating residual FDG-avid sites after a T-cell-based therapy has already acted, in an attempt to eliminate the remaining disease [18,27]. Here, too, however, direct BsAb-specific data are lacking; the hypothesis remains extrapolated from CAR-T experience, not demonstrated in the BsAb setting (Section 3.1).

In both scenarios, what remains valid is that CAR-T and BsAbs alike depend on an adequate T-cell compartment, infused in the former and endogenous in the latter, for their therapeutic activity. Whether RT-induced lymphopenia is consequential for the endogenous T-cell compartment of BsAb-treated patients, in the way inadequate lymphodepletion can be for CAR-T engraftment, remains itself unestablished. The first available data on this question are discussed in Section 3.2 and should not be assumed by analogy.

## 4. Discussion and Future Directions

CD20×CD3 bispecific antibodies have transformed the treatment of relapsed/refractory B-cell lymphoma but remain a non-curative option for most patients: a substantial proportion do not respond, and even among responders, disease control is often not sustained. This unmet clinical need motivates interest in combining BsAb therapy with other treatment modalities.

Radiotherapy has long been a cornerstone of lymphoma treatment across multiple clinical contexts, reflecting the marked radiosensitivity of these malignancies. Beyond its direct cytocidal effect, its immunomodulatory potential has been increasingly recognised. This phenomenon has been explored primarily in solid tumours, but there is emerging evidence in lymphoma itself, including abscopal-like regressions after involved-site RT in indolent disease [25]. These properties make RT a natural candidate for the integration with newer immune-based treatment paradigms. This role is already established alongside CAR-T therapy and is now beginning to emerge alongside BsAbs. This account is necessarily incomplete, however: as detailed in Section 3.1, RT could equally work against BsAb activity, by reducing the CD20 antigenic target, depleting intratumoral or circulating T cells, damaging draining lymphoid structures, or inducing immunosuppressive myeloid populations. These antagonistic possibilities have received markedly less study than the hypothesised benefits. We see no basis in the current evidence for weighting one direction over the other.

The available reports indicate feasibility without a uniform safety signal but say little about optimal integration. The biological mechanisms that might underlie a therapeutic synergy remain exploratory and hypothesis-generating and do not currently constitute established clinical indications.

This biological plausibility, however, is precisely why the question deserves a dedicated prospective investigation rather than dismissal. RT is a treatment modality already central to lymphoma management and is now shown to be feasible alongside BsAb therapy in early experience. It merits a genuine, trial-based answer on whether it can be integrated safely and with a benefit.

Several open questions, discussed throughout this review, deserve prospective, comparative, and translational study; none should be read as ranked above the others: (i) whether RT-induced lymphopenia meaningfully affects BsAb efficacy, and through which T-cell compartments; (ii) whether RT-mediated cytoreduction actually reduces CRS/ICANS incidence or severity, beyond the established baseline association between tumour burden and CRS/ICANS risk, now being tested in NCT06867536 and NCT07634289; (iii) whether the comprehensive irradiation of residual PET-avid disease after a partial BsAb response improves outcomes, and how residual viable lymphoma can be reliably distinguished from post-treatment inflammation or flare before such irradiation is undertaken; and (iv) the potential antagonistic biological effects of RT on the BsAb mechanism, including target-antigen loss, T-cell depletion, immunosuppressive myeloid induction, TGF-β signalling, and antigen-low clone selection, which have received comparatively little study to date.

As BsAbs move into earlier treatment lines and combination strategies, the role of RT may evolve, but only prospective, comparative data can establish whether it does, and how. We make no recommendation for or against RT in this setting; until prospective data exist, what can be said is only descriptive. Where RT is used, for symptomatic disease, suspected tumour flare (with appropriate caution given the discordant case-level evidence), or oncological emergencies, current practice treats it as an individualised, case-by-case decision taken by a multidisciplinary board bringing together radiation oncology and haematology expertise, not as a planned or standardised component of treatment outside this context. Whether that practice pattern is the right one is itself one of the open questions in Section 5.

## 5. Research Hypotheses and Priorities for Future Study

The four clinical situations below are presented as open research questions requiring prospective, comparative study, not as a treatment algorithm and not as recommendations. RT use in each situation should be regarded as an individualised, off-protocol clinical decision (Figure 2).

• Bulky/high-risk disease at BsAb initiation: Does RT-mediated cytoreduction actually lower the CRS/ICANS incidence or severity, beyond the known baseline association between tumour burden and risk? This is now being tested in NCT06867536 and NCT07634289.
• Apparent early progression (possible tumour flare reaction): Does RT safely allow BsAb therapy to continue after a biopsy or imaging confirmation, or can RT itself trigger a CRS-like reaction?
• Residual PET-avid disease after a partial BsAb response: Does the residual avidity reflect viable lymphoma or post-treatment inflammation, and does comprehensive RT improve outcomes compared with focal or no RT?
• Oncological emergency during BsAb step-up dosing: Is BsAb antitumour activity actually delayed during step-up dosing, or does step-up dosing simply manage the CRS risk without delaying the antitumour effect?

## 6. Limitations of This Review

This review has several limitations, beyond those already noted at each relevant point above. Embase, Scopus, and Web of Science were not searched, for resource/access reasons. The post hoc verification search reduces, but does not eliminate, this gap, and non-English-language reports may have been missed. The evidence base is highly susceptible to publication bias, as favourable experiences are more likely to be reported than unsuccessful or toxic RT–BsAb combinations; the newly identified adverse case report [17] is a useful, but isolated, counter-example. The reviewed sources combine aggressive and indolent lymphomas, different bispecific constructs, treatment lines, RT indications, and radiation schedules, generally without accounting for prior CAR-T therapy, baseline lymphopenia, tumour burden, or disease distribution. RT was also preferentially used in symptomatic, bulky, or high-risk patients, a form of confounding by indication that likely differs systematically from the overall BsAb-treated population. Under this heterogeneity, aggregate statements about feasibility, safety, or efficacy have limited interpretative value, and we have tried throughout this review to avoid making them. Several of the proposed research questions, particularly those concerning consolidative RT and the comprehensive treatment of residual disease, are extrapolated from the CAR-T experience rather than directly demonstrated in CD20×CD3 lymphoma; this extrapolation should not be mistaken for direct evidence. As this is a Perspective rather than a systematic or scoping review, no protocol registration was applicable. Taken together, the available evidence should be interpreted as hypothesis-generating and should not inform routine clinical decision-making.

## 7. Conclusions

This Perspective reviews a small, heterogeneous, and partly discordant body of direct evidence on combining radiotherapy with CD20×CD3 bispecific antibodies in B-cell lymphoma. RT has occasionally been delivered around BsAb therapy without a consistent toxicity signal, but it establishes neither clinical benefit, synergy, nor optimal sequencing. The four clinical scenarios discussed in Section 5 should be read as research questions, not practice guidance. Prospective comparative trials and translational studies of RT-induced lymphopenia and potential antagonistic biological effects are needed before this combination can be considered beyond an individualised, off-protocol clinical decision.

## Figures and Tables

**Table 1:** (a) Study characteristics of identified evidence on RT combined with CD20×CD3 bispecific antibodies in B-cell lymphoma, classified by evidentiary tier. Blinatumomab (CD19×CD3) and multiple myeloma data are labelled and are not treated as CD20×CD3-lymphoma evidence. (b) Treatment parameters and outcomes for the same sources (rows correspond to Table 1a). RT dose/fractionation and toxicity are reported as stated in the source; entries marked “not reported” reflect genuine gaps in current reporting, not omissions by the authors.

(**a**)							
**Source**	**Tier**	**n (CD20×CD3 Lymphoma Specifically)**	**Lymphoma Subtype**	**Prior Lines**	**BsAb Agent(s)**		**RT Target/Site/Volume**
Loap et al., Strahlenther Onkol 2024 [8]	Direct	1	DLBCL	Multiple (post CAR-T)	Glofitamab		Single early-progressing site (VMAT)
Fujita et al., Int J Hematol 2025 [9]	Direct	1	DLBCL	Not specified	Epcoritamab		Cervical nodal TFR site
*New:* RT-triggered CRS-compatible fever case, 2026 [17]	Direct	1	DLBCL	Not specified (late-phase therapy)	Epcoritamab		Abdominal lymph node
Ren et al., Blood 2025 (ASH abstract) [15]	Mixed/indirect	12 (RT not disaggregated by partner)	Not fully specified	Not fully specified	Glofitamab		Not disaggregated by combination partner
Ababneh et al., Radiother Oncol 2026 [10]	Direct for population/exposure (LBCL subgroup); mixed/indirect for outcomes (response, CRS, ICANS not disaggregated by histology in source)	7 of 26 (19/26 multiple myeloma, indirect only)	LBCL (subgroup); myeloma (remainder, indirect)	Heterogeneous	Glofitamab, epcoritamab (LBCL); elranatamab, teclistamab, talquetamab (myeloma, indirect)		45 irradiated sites overall (43 evaluable for response); not further specified by subgroup
Baron et al., Clin Lymphoma Myeloma Leuk 2026 [16]	Direct for CRS/ICANS (disaggregated by agent); mixed/indirect for ORR (not disaggregated by agent in source)	29 pts/31 courses; ~77% CD20×CD3 courses, ~23% blinatumomab (indirect)	B-NHL, incl. LBCL and follicular lymphoma	Heterogeneous, multiple prior lines	Mosunetuzumab (61%), blinatumomab (23%, indirect), epcoritamab, glofitamab		52 irradiated sites
NCT07528352 (Univ. of Utah) [12]	Ongoing trial, no results	Estimated *n* = 12	R/R DLBCL	Not yet reported	Epcoritamab/Glofitamab		Not yet reported
NCT06867536 (China) [13]	Ongoing trial, no results	Estimated per protocol	R/R DLBCL, high tumour burden	Not yet reported	Glofitamab		Not yet reported
*New:* NCT07634289 “Radio-GLO” [14]	Not yet recruiting (verified June 2026); no results	Estimated *n* = 40	R/R DLBCL	Not yet reported	Glofitamab (±obinutuzumab)		Not yet reported
Strati & Spiotto, Lymphatics 2023 [11]	Background only	N/A	N/A (narrative review)	N/A	Various (checkpoint inhibitors + bispecifics)		N/A
(**b**)							
**Source**	**RT dose/fractionation**	**Timing vs. BsAb**	**Pre-/post-RT lymphocyte data**	**Follow-up**	**Response (lesion- vs. patient-level)**	**PFS/OS**	**Toxicity**
Loap et al. [8]	Not specified	Concurrent	Not reported	3 months	Lesion-level: complete metabolic response	Not reported	Mild para-spinal pain; no pulmonary toxicity
Fujita et al. [9]	Not specified	During (post biopsy-confirmed TFR)	Not reported	Not specified	Lesion-level: local control of TFR; BsAb continued	Not reported	Not reported
RT/CRS fever case [17]	40 Gy/20 fractions	During (cycle 8, late-phase)	Not reported	Short-term (index episode)	Not the focus (safety report)	Not reported	CRS-compatible fever (grade 1 ASTCT) after RT; resolved with IL-6 blockade
Ren et al. [15]	Not disaggregated	Variable, per clinical practice	Not reported	Not specified	Not disaggregated by combination partner	Not reported	Not disaggregated by combination partner
Ababneh et al. [10]	Heterogeneous, not further specified	Before, during, or after BsAb	Not reported	Not specified	Local control and symptomatic response in all treated sites	Not reported	Manageable regardless of RT sequencing
Baron et al. [16]	Median 20 Gy (range 4–45 Gy), heterogeneous	Before (50%), during (13%), after (37%)	Early post-RT lymphopenia common; not assoc. with RT timing	Not uniformly specified	In-field ORR 84% (CR 45%, PR 39%) among 44 evaluable lesions	Not reported	All RT-related toxicities grade 1–2; no RT-related hospitalisations; CRS 6/24 (25%), ICANS 2/24 (8.3%) among CD20×CD3 courses (mosunetuzumab, epcoritamab, glofitamab), disaggregated from source data; blinatumomab courses reported separately (CRS 1/7, ICANS 2/7) and excluded from these figures
NCT07528352 [12]	5 fractions (total dose not yet reported)	Before BsAb initiation	Not yet reported	Recruiting (June 2026)	Safety/tolerability endpoints (no results)	Not yet reported	Not yet reported
NCT06867536 [13]	Hypofractionated; regimen not yet reported	Before glofitamab (cytoreductive)	Not yet reported	Est. completion 2028	Not yet reported	Not yet reported	Not yet reported
NCT07634289 [14]	Not yet reported	Combined with glofitamab; molecular-imaging endpoints	Not yet reported	Est. completion 2032	Phase II, not yet reported	Not yet reported	Not yet reported
Strati & Spiotto [11]	N/A	N/A	N/A	N/A	Bispecifics addressed only as a subsection within a wider review	N/A	N/A

## Data Availability

No new data were generated or analysed. All sources discussed are publicly available and cited in the reference list. Database-specific search terms and dates (Supplementary Table S1) are provided as Supplementary Materials.

## Supplementary Materials

The following supporting information can be downloaded at: https://www.mdpi.com/article/10.3390/cancers18172895/s1, Figure S1: PRISMA-ScR flow diagram of study identification and selection; Table S1: Electronic search strategies.
