# Supplementary material for: Radiotherapy in Combination with CD20×CD3 Bispecific Antibodies for B-Cell Lymphoma: Biological Rationale, Current Evidence, and Open Questions
Source: Cancers (Basel). 2026 Sep 7;18(17):2895. doi: 10.3390/cancers18172895 (PMC13565725; doi:10.3390/cancers18172895)
Supplement: Supplementary file 1 [file cancers-18-02895-s001.zip › Figure_S1_PRISMA_ScR_flow_diagram.pdf]

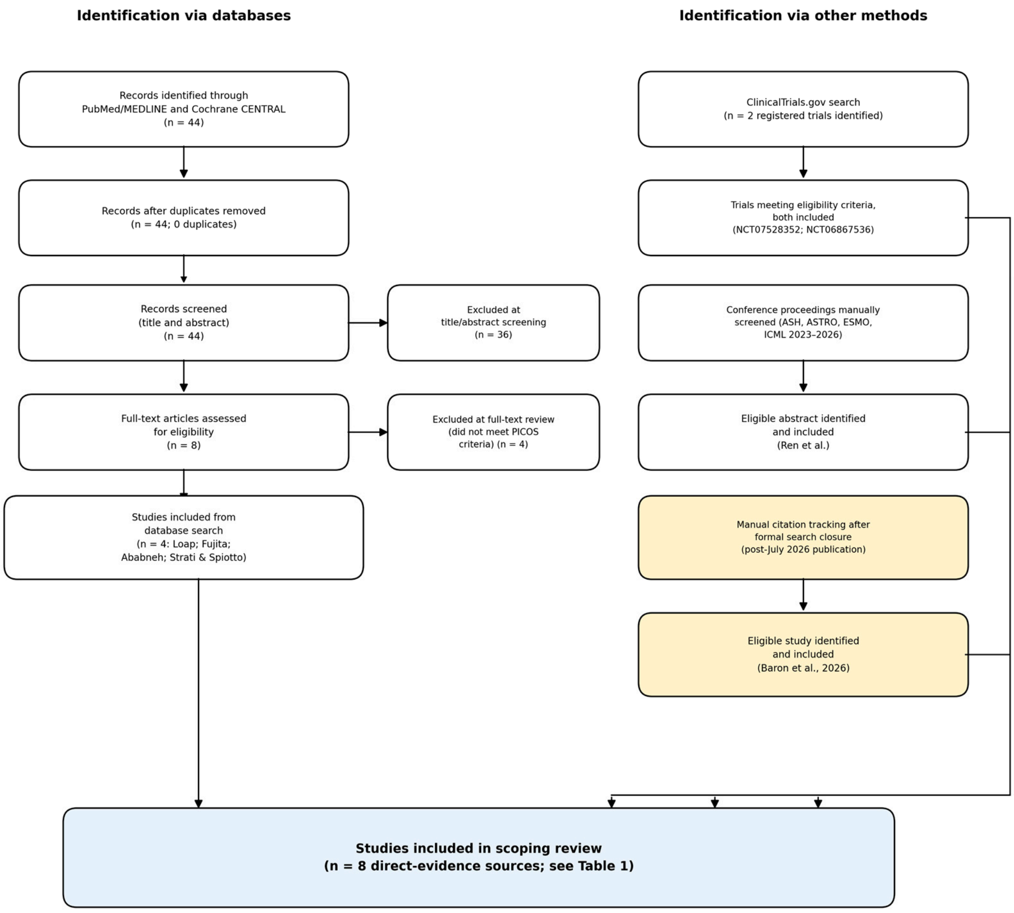

Figure S1. PRISMA-ScR flow diagram of study identification and selection.

One additional record (Baron et al., 2026) was identified through manual citation tracking after the formal electronic search date and is included alongside the seven records retrieved by the structured search.

Figure S1. PRISMA-ScR flow diagram of study identification and selection.
