# Supplementary material for: Radiotherapy in Combination with CD20×CD3 Bispecific Antibodies for B-Cell Lymphoma: Biological Rationale, Current Evidence, and Open Questions
Source: Cancers (Basel). 2026 Sep 7;18(17):2895. doi: 10.3390/cancers18172895 (PMC13565725; doi:10.3390/cancers18172895)
Supplement: Supplementary file 1 [file cancers-18-02895-s001.zip › Table_S1_Electronic_Search_Strategies.pdf]

Table S1. Electronic search strategies.

**Database**	**Search strategy**	**Date searched**
PubMed/MEDLINE	("Radiotherapy"[Mesh] OR radiotherapy OR radiation therapy OR irradiation OR radiation) AND ("Bispecific Antibody"[Mesh] OR bispecific antibod* OR T-cell engager* OR T cell engager* OR CD20×CD3 OR CD20-CD3 OR epcoritamab OR glofitamab OR mosunetuzumab OR odronextamab) AND ("Lymphoma"[Mesh] OR lymphoma OR B-cell lymphoma OR diffuse large B-cell lymphoma OR DLBCL OR follicular lymphoma)	July 2026
Cochrane CENTRAL	(Radiotherapy OR "radiation therapy" OR irradiation OR radiation) AND ("bispecific antibod*" OR "T-cell engager*" OR "T cell engager*" OR CD20×CD3 OR CD20-CD3 OR epcoritamab OR glofitamab OR mosunetuzumab OR odronextamab) AND (lymphoma OR "B-cell lymphoma" OR DLBCL OR "diffuse large B-cell lymphoma" OR "follicular lymphoma")	July 2026
ClinicalTrials.gov	Condition/disease: lymphoma OR B-cell lymphoma OR diffuse large B-cell lymphoma OR follicular lymphoma. Other terms: radiotherapy OR radiation therapy AND bispecific antibody OR CD20×CD3 OR epcoritamab OR glofitamab OR mosunetuzumab OR odronextamab	July 2026
